# Clinical characteristics and care pathways of patients with personality disorder who died by suicide

**DOI:** 10.1192/bjo.2020.11

**Published:** 2020-03-18

**Authors:** Sandra Flynn, Jane Graney, Thabiso Nyathi, Jessica Raphael, Seri Abraham, Sandeep Singh-Dernevik, Alyson Williams, Nav Kapur, Louis Appleby, Jenny Shaw

**Affiliations:** Division of Psychology and Mental Health, Faculty of Biology, Medicine and Health, School of Health Sciences, University of Manchester, UK; Greater Manchester Mental Health NHS Foundation Trust, UK; Pennine Care NHS Foundation Trust, UK

**Keywords:** Personality disorder, suicide and self-harm, mental health services

## Abstract

**Background:**

It is estimated that 1 in 10 people have a personality disorder. People with emotionally unstable personality disorder are at high risk of suicide. Despite being frequent users of mental health services, there is often no clear pathway for patients to access effective treatments.

**Aims:**

To describe the characteristics of patients with personality disorder who died by suicide, examine clinical care pathways and explore whether the care adhered to National Institute for Health and Care Excellence guidance.

**Method:**

National consecutive case series (1 January 2013 to 31 December 2013). The study examined the health records and serious incident reports of patients with personality disorder who died by suicide in the UK.

**Results:**

The majority had a diagnosis of borderline/emotionally unstable or antisocial personality disorder. A high proportion of patients had a history of self-harm (*n* = 146, 95%) and alcohol (*n* = 101, 66%) or drug misuse (*n* = 79, 52%). We found an extensive pattern of service contact in the year before death, with no clear pathway for patients. Care was inconsistent and there were gaps in service provision. In 99 (70%) of the 141 patients with data, the last episode of care followed a crisis. Access to specialised psychological therapies was limited; short-term in-patient admissions was adhered to; however, guidance on short-term prescribing for comorbid conditions was not followed for two-thirds of patients.

**Conclusions:**

Continuity and stability of care is required to prevent, rather than respond to individuals in crisis. A comprehensive audit of services for people with personality disorder across the UK is recommended to assess the quality of care provided.

People with personality disorder exhibit a complex set of behaviour patterns characterised by instability of mood and impaired social functioning.^1^ The presence of personality disorder can increase the risk of certain adverse behaviours including violence and self-harm. In a study examining mortality by suicide or undetermined cause, personality disorder was found to be the diagnostic category with the highest risk in women, an increase of over 20-fold.^2^ The evidence suggests that patients with borderline personality disorder are high users of mental health services compared with other patients.^3^ This can lead to difficulties in relationships between staff and patient, particularly if patients exhibit challenging behaviour. The high use of services has been linked to the psychopathology of borderline personality disorder, but also attributable to comorbid diagnoses,^4^ particularly substance misuse, which is a recognised criterion for excluding patients from specialist services.^5^ Guidance issued by the National Institute for Health and Care Excellence (NICE) outlines quality standards for the improvement of care and recommend that psychological interventions are made widely available, with appropriate patient involvement in choosing the type, intensity and duration of therapies.^6^ The guidelines also recommend the short-term use of medication as part of the overall treatment plan and the avoidance of in-patient care where possible, as this has been shown to be less effective and often counterproductive.^6–8^ However, increasing demand for time-intensive specialist therapies, coupled with a lack of resources has limited their accessibility.

In this UK study we aimed to (a) describe the sociodemographic and clinical features of patients with personality disorder who died by suicide and had been in recent contact with mental health services; (b) examine the clinical care pathway experienced by patients prior to their deaths and; (c) examine the extent to which care received by patients adhered to NICE guidelines for borderline and antisocial personality disorder.

## Method

### Research design

The National Confidential Inquiry into Suicide and Safety in Mental Health (NCISH) collate and manage a UK-wide consecutive case series of people who have died by suicide while under the care of mental health services, within 12 months of their death. The present study is a retrospective study. It involved examination of the files of patients’ in the NCISH database dated between 1 January 2013 and 31 December 2013. Medical records were received for 138 individuals and serious adverse incident reports on 102. Overall, information (medical records and/or serious adverse incident reports) was received on 142 out of 154 patients, a response rate of 92%.

### Data collection

Data collection had two main stages. First, using the NCISH database, we identified patients who died by suicide during the study period. The NCISH database is comprehensive and contains information on sociodemographic characteristics, clinical history, aspects of care and antecedents of the suicide. Questionnaires were completed by the consultant psychiatrist responsible for the patient's care. Information on the NCISH methodology has previously been published and described in detail elsewhere.^9^ Second, copies of the deceased's medical records were obtained from National Health Service (NHS) trusts or health boards. We requested records for the 12 months prior to the date of death only. We also requested copies of serious incident/critical incident review/serious adverse incident reports. Serious adverse incident reports provided a detailed account of the organisation's internal investigation of the death, often using root cause analysis. The reports included timelines of care, staff interviews and recommendations for improving patient safety. Data from the medical records was systematically extracted using a proforma designed by the authors.

### Inclusion criteria

DSM-5 was used to define the personality disorder categories.^10^ Patients who died by suicide were included if they had a primary diagnosis of personality disorder or a secondary diagnosis of personality disorder, where the primary diagnosis was alcohol misuse/dependence or drug misuse/dependence. The comparison group was all patient suicides within the time period with ‘other primary diagnoses’. Because of the level of comorbidity in personality disorder populations and the potential for misclassification bias in the comparator, we ran a sensitivity analyses to test validity using patients with a primary diagnosis of affective disorder only (with no comorbid personality disorder). When comparing demographic, behavioural and clinical characteristics and contact with mental health services, the only differences (where affective disorder was the comparator compared with all patient suicide) was that patients with affective disorder were less likely to be unmarried and live alone. For those whose first contact with services was between 1 and 5 years before the suicide, there was no difference compared with affective disorder, but a significant difference compared with all patient suicides. Overall, the results of this *post hoc* analysis suggests that using all patient suicides as comparator has not affected the results.

### Definitions

We defined the last episode of care as a distinct event in the patient's treatment history. This involves a single last contact or a sequence of unbroken care, even if this was from a number of services (i.e. accident and emergency, in-patient, crisis resolution and home treatment, primary care). The potential lethality of self-harming prior to the suicide was recorded from the incident description recorded in the case notes.

### Patient and public involvement

No patients or members of the public were involved in developing the research question, design or implementation of the study. We did not invite the public or patients to advise on interpretation or writing up of results. However, the NCISH independent advisory group provides external oversight and includes lay members and representatives from key stakeholder groups.

### Ethics

All procedures contributing to this work comply with the ethical standards of the relevant national and institutional committees on human experimentation and with the Helsinki Declaration of 1975, as revised in 2008. Approvals were sought and received from the National Research Ethics Service Committee North West (NRES) (31 March 2016); Health Research Authority Confidential Advisory Group (HRA-CAG) (amendment to existing approval) (31 March 2016); Public Benefit and Privacy Panel for Health and Social Care (PBPP) (6 July 2016); and Research Management and Governance approvals from individual NHS trusts. We obtained exemption under section 251 of the NHS Act 2006 (formerly section 60 of the Health and Social Care Act 2001) enabling access to confidential and identifiable information without informed consent in the interest of improving patient care.

### Statistical analysis

Stata 13 was used to analyse the data.^11^ The descriptive statistics such as frequency analysis and chi-squared were used. The Pearson's chi-squared tests were used to examine the associations between the subgroups. Cell counts under three were not reported due to confidentiality reasons. Missing data were excluded. All proportions are provided as valid percentages.

## Results

### Characteristics of patients with personality disorder who died by suicide

#### Social and behavioural characteristics

We were notified of 1601 patients who died by suicide between 1 January and 31 January 2013 in the UK. Of these, 154 (10%) people had a diagnosis of personality disorder. In 132 (86%), personality disorder was the primary diagnosis, the remaining 22 had a secondary diagnosis, where the primary diagnosis was alcohol (*n* = 14) or substance misuse/dependence (*n* = 8). Information was available on the type of personality disorder for 128 individuals. The majority had a diagnosis of borderline/emotionally unstable 116 (91%) or antisocial personality disorder (*n* = 5, 4%). The remaining seven patients (5%) had dependent, histrionic, paranoid, schizoid or mixed personality disorder.

Table 1 shows the sociodemographic characteristics of patients with personality disorder compared with patients with other diagnoses. A total of 55% of the 154 patients who died by suicide were female, a significantly higher proportion compared with other mental health patients. The mean age of all patients at the time of death was 42 years. Alcohol and drug misuse, a previous history of violence and self-harm were also more common among patients with personality disorder.

**Table 1:** Sociodemographic characteristics of patients with personality disorder who died by suicide compared with patients with other diagnoses

	Personality disorder (*n* = 154)	Other diagnoses^a^ (*n* = 1447)	*P*
Gender, *n* (%) 95% CI for %			
Male	69/154 (45) 37–53	1030/1447 (71) 69–74	<0.01
Female	85/154 (55) 47–63	417/1447 (29) 26–31	<0.01
Age, years: median (range)	42 (17–82)	47 (12–96)	<0.01
Not currently married, *n* (%) 95% CI for %	119/152 (78) 71–85	1004/1400 (72) 69–74	0.09
Living alone, *n* (%) 95% CI for %	82/149 (55) 47–63	689/1377 (50) 47–53	0.25
Unemployed/sickness leave, *n* (%) 95% CI for %	121/148 (82) 75–88	813/1382 (59) 56–61	<0.01
Black, Asian and minority ethnic group, *n* (%) 95% CI for %	9/152 (6) 3–11	95/1426 (7) 5–8	0.73
Method of suicide, *n* (%) 95% CI for %			
Self-poisoning	61/154 (40) 32–48	356/1446 (25) 22–27	<0.01
Hanging	58/154 (38) 30–46	643/1446 (44) 42–47	0.11
Jumping	15/150 (10) 6–16	222/1446 (15) 14–17	0.06
History of alcohol misuse, *n* (%) 95% CI for %	101/152 (66) 58–74	663/1409 (47) 44–50	<0.01
History of substance misuse, *n* (%) 95% CI for %	79/151 (52) 44–60	512/1401 (37) 34–39	<0.01
History of violence, *n* (%) 95% CI for %	62/150 (41) 33–50	295/1384 (21) 19–24	<0.01
History of self–harm, *n* (%) 95% CI for %	146/154 (95) 90–98	873/1391 (63) 60–65	<0.01

a. Other diagnoses include schizophrenia and other delusional disorders, affective disorder, alcohol dependence/misuse, drug dependence misuse, adjustment disorder, anxiety disorder, eating disorder, dementia, intellectual disability, pervasive developmental disorder/autistic spectrum disorder and attention-deficit hyperactivity disorder/conduct disorder.

In the 3 months before their death, 60% reported problems with alcohol and 46% with drug misuse. In total, 95% of patients had a history of self-harm. The last episode of self-harm occurred within a week of their death in 22 patients (18% of the 122 with data). Repeated incidents of self-harm were common in the year before suicide (*n* = 79 of 118, 67%). During the last reported episode of self-harm, the most common methods recorded were self-poisoning with drugs (*n* = 81 of 127, 64%) and cutting (*n* = 37 of 132, 28%). In 32 of 110 (29%) the potential lethality of this act was judged to be high. The nature of self-harm was more commonly reported to be impulsive (*n* = 55 of 72, 76%) rather than premeditated (*n* = 11 of 69, 16%).

There were 116 of 117 patients (99%) who had an adverse life event prior to suicide recorded in the case notes, most commonly relationship problems with intimate partners, family members and friends, difficulties with accommodation, financial problems and substance misuse. The most common method of suicide was self-poisoning. This was significantly higher among patients with personality disorder; hanging was a more commonly used method among other patients (Table 1). Opiates (heroin, methadone) were the most frequently used drug in fatal overdose (*n* = 19 of 60, 32%), followed by antipsychotics (*n* = 12, 20%). Of all drugs used in the fatal overdose (including analgesics), 20 patients used drugs that had been prescribed for them, 4 used drugs prescribed for someone else, 12 used non-prescribed drugs.

#### Clinical characteristics

Patients’ clinical characteristics are shown in Table 2. There were a number of clinical differences between patients with personality disorder and other patients who died by suicide. In-patient deaths were uncommon; however, patients with personality disorder were more likely to have taken their own life after being recently discharged from in-patient care. The last admission was more likely to be a readmission within 3 months of being discharged from hospital. The majority of admissions were brief (*n* = 44 of 100, 44%) less than 7 days, with 4 of 100 (4%) more than 13 weeks.

**Table 2:** Clinical characteristics of patients with personality disorder who died by suicide compared with patients with other diagnoses

	Personality disorder (*n* = 154)			Other diagnoses^a^ (*n* = 1447)			
	*n*	%	95% CI	*n*	%	95% CI	*P*
In-patients	7/154	5	2–9	84/1446	6	5–7	0.52
Recent (<3 months) discharge	31/145	21	15–29	170/1354	13	11–14	<0.01
Missed last contact	39/145	27	20–35	345/1335	26	24–28	0.78
Non-adherence with medication in last month	16/143	11	7–18	143/1304	11	9–13	0.94
Duration of illness							
Less than 12 months	4/153	3	1–7	300/1368	22	20–24	<0.01
More than 5 years	125/153	82	75–87	698/1368	51	48–54	<0.01
Over five previous admissions	29/153	19	13–26	104/1419	7	6–9	<0.01
First contact with services							
Less than 12 months	12/154	8	4–13	358/1447	25	23–28	<0.01
1–5 years ago	36/150	24	17–31	382/1415	27	25–30	<0.01
More than 5 years	104/153	68	60–76	668/1421	47	45–50	<0.01
Last admission was a readmission after 3 months	22/103	21	14–31	86/669	13	10–16	0.02
Last contact within 7 days of death/offence	72/154	47	39–55	656/1429	46	43–49	0.84
Symptoms of mental distress at last contact	111/146	76	68–83	855/1389	62	59–64	<0.01

a. Other diagnoses include schizophrenia and other delusional disorders, affective disorder, alcohol dependence/misuse, drug dependence misuse, adjustment disorder, anxiety disorder, eating disorder, dementia, intellectual disability, pervasive developmental disorder/autistic spectrum disorder and attention-deficit hyperactivity disorder/conduct disorder.

Two-thirds had their first contact with services more than 5 years before taking their own life, whereas patients with other diagnosis were more likely to have had recent contact with services prior to suicide. Nearly half had their last contact with services within a week of their death. At last contact 44 of 142 (31%) had symptoms of depression and 34 of 142 (24%) evidence of suicidal ideation. All those who presented with suicidal ideation had a history of self-harm. The risk of the self-harm episode being lethal was considered to be moderate or high in 13 of 25 individuals (52%).

### Pathways into care

#### Care pathway at last episode

**Fig. 1:**
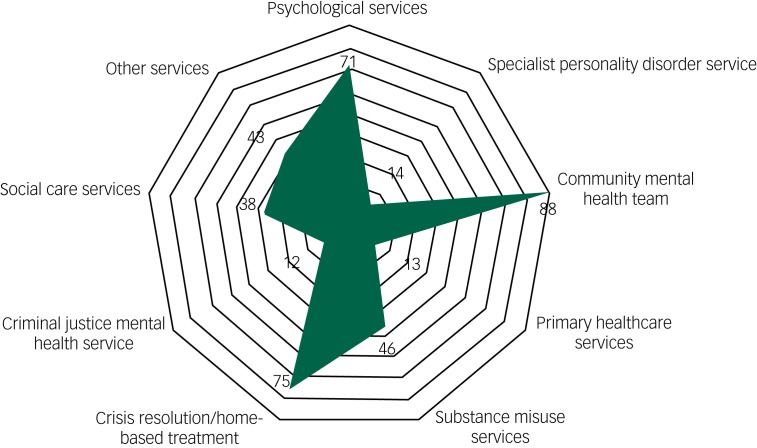
The pattern of referral the year before the patient's death. The figures are the number of times (*n*) patients had been referred to services the year before their death (of a total of 113 patients with data).

Patients had frequently accessed a large range of services within a year of their death. The mean number of contacts with any mental health service was 27 (range 1–313). Twenty-one of 140 patients (15%) had more than 50 contacts with services. The overall pattern of referral in the year before death can be seen in Fig. 1.

In the 141 individuals with data, the last episode of care before the patient's suicide most commonly followed a crisis (*n* = 99, 70%) rather than a routine appointment (*n* = 42, 30%). Of those assessed following a crisis, 83 of 97 (86%) were referred into mental health services. The main reason for the referral was because of the risk of harm to themselves (*n* = 77 of 97, 79%). Overall, 51 of 115 (44%) were referred into a community mental health team and 36 of 116 (31%) into a crisis resolution/mental health home treatment team.

During the last contact with services 19 of 136 patients (14%) were assessed and then not referred into mental health services, either because they took their own life prior to full assessment or that they were judged to have no mental health service needs. Furthermore, a smaller group of patients had disengaged from services, 39 of 145 (27%) missed their last contact and 16 of 143 (11%) were non-adherent with treatment in the month before their death.

### Adherence to NICE guidance

#### Access to psychological therapies

In total, 24 of 100 patients (24%) had previously been referred to specialist personality disorder services but were not currently under that service. In the year before their death, 14 of 140 (10%) had been referred, 9 patients attended. Most patients had been offered psychological therapy at some time during their contact with mental health services (*n* = 84 of 109, 77%) and 53 of 98 (54%) received that psychological therapy. The most common therapeutic intervention received was cognitive–behavioural therapy (CBT, *n* = 16 of 107, 15%) followed by dialectical behavioural therapy (DBT, *n* = 15 of 100, 15%). The number of patients receiving mentalisation-based therapy (MBT) was small (*n* = 3, 3%). A wide range of other therapies were offered to patients (*n* = 24 of 100, 24%) including anxiety management, art therapy, cognitive remediation therapy, eye movement desensitisation and reprocessing, group therapy, mindfulness, schema therapy, sexual assault therapy, trauma services and transference-focused psychotherapy.

#### Prescribing medication

Medication had been used as part of the treatment plan at some time during their contact with mental health services in 137 of 138 (99%) patients. In 59 of 94 patients (63%) the prescribing did not follow NICE guidance with regard to short-term prescribing. In the year before the suicide, 111 of 142 were prescribed antidepressant medication (78%), 79 of 141 were prescribed antipsychotic medications (56%).

#### Risk assessment

During the last episode of care, a risk assessment for suicide, self-harm and/or violence was undertaken in 104 of 116 (90%), a risk formulation undertaken in 79 of 107 (74%) and risk management plan developed in 73 of 101 (72%). At last contact, the immediate risk of suicide was judged to be low or no risk in 96 of 136 (71%). The long-term risk was considered to be moderate or high in 102 of 134 (76%).

## Discussion

### Main findings

The patients in this study were most commonly diagnosed with borderline personality disorder and had a long history of illness and complex needs. Almost all had a history of self-harm, commonly involving overdose. Almost three-quarters had been recently seen by services following an incident of self-harm. Half had their last service contact within a week of their deaths. The study suggests that the standard of treatment and care recommended by NICE was not being consistently received by patients with personality disorder, prior to their death.

### Pattern of contact with mental health services

The pattern of contact and extensive use of mental health services by patients in our study is consistent with the findings of previous research.^3^ Patients with borderline personality disorder are often well known to services as they have a long history of illness. In a study of the health costs of patients with personality disorder, researchers found a high use of psychiatric, ambulance and accident and emergency services mainly in crisis (but this was also true of patients with major depression).^12^ Our finding show the route into mental healthcare for people with borderline personality disorder was unclear, with multiple contacts with a wide range of services.

The current configuration of mental health services was not designed for patients with personality disorder. New crisis services were introduced via the Mental Health Act 2007 for people with serious mental illness such as schizophrenia, as short-term interventions. However, in our qualitative study, patients with personality disorder reported a lack of early intervention and support during an episode. Patients felt that if they had regular contact with services, they would less likely be at risk of self-harm.^13^ This finding is consistent with previous research that found that those with no community care had an elevated risk of suicide.^14^ Although it may not be feasible in the current economic climate to redesign a service for personality disorder, consideration should be given to offering regular appointments and increasing NHS day care services, which have been shown to have improved outcomes.^15,16^ The evidence from this study suggests services could be improved by providing consistency and continuity of care, regular support to break the cycle of crisis contact and rejection and referral into specialist personality disorder services as part of a long-term treatment plan.

### Psychological interventions

The NICE guidance for the recognition and treatment of personality disorder published almost a decade ago recommended avoiding hospital admissions, providing evidence-based psychological interventions and using pharmacological approaches as a adjunctive short-term measure, specifically to manage comorbid diagnoses.^6,7^ We found that although almost half of the patients had been admitted to in-patient care, this was usually for a brief admission; only 4% of admissions lasted more than 13 weeks. However, the guidance on treatment was less consistently adhered to. Psychosocial treatment of more than 3 months duration is the primary approach recommended by NICE for people with borderline personality disorder.

We found few patients had received treatments such as DBT and MBT prior to their death. These therapeutic interventions are intense psychological treatment delivered by specialist practitioners who have received extensive training. They are resource intensive, expensive to run and in high demand from patients, who may not meet the criteria. We found (perhaps as an alternative) CBT was offered to a large proportion of patients in this study. There is strong empirical support for CBT and CBT-based psychological treatments for borderline personality disorder.^17^ Davidson et al (2006) found CBT used in conjunction with treatment as usual to be helpful in reducing acts of suicide.^18^ The efficacy and effectiveness of psychotherapy is less clear, although it is considered to be cost-effective for the short-term treatment of cluster C avoidant, dependent and obsessive–compulsive personality disorder.^19^ Treatments such as DBT (recommended for women with reoccurring self-harm) and MBT are largely provided by specialist services, which limits availability. However, an integrated approach to generating a treatment model that combines effective methods across the range of therapies in order to ‘accommodate the extensive heterogeneity of borderline personality disorder and its complex aetiology’ has been proposed.^20^ This approach would potentially make these services more accessible to a greater number of patients in the future, without having an impact on already stretched resources.

### Implications

The results of this study suggest a more flexible approach and understanding of individual patients is needed. Currently, comorbid mental health diagnoses such as, anxiety, depression and substance misuse complicate treatment pathways. Although they can open the doors to services, patients can feel ‘batted’ around in a cycle of referrals and unable to access therapies that could address the underlying trauma and distress. It has been suggested that outcomes could be improved by applying more targeted interventions delivered over a short period of time as part of a longer management plan, rather than an intensive therapy.^21^ The interventions can be adapted for easier use in a wider range of settings. The researchers also recommended the use of pharmacotherapy, which should be time limited for the management of specific symptoms and ‘withdrawn when these are resolved’.

Our findings show that the use of pharmacotherapy for personality disorder was commonplace. We found almost half of the patients were being prescribed benzodiazepines in the year before their deaths, often in conjunction with other medication. Prescribing benzodiazepines is not recommended for patients with personality disorder. Benzodiazepines have been found to reduce inhibitions therefore worsening impulsivity, increasing suicidal ideation and reducing behavioural control.^22^ Although progress has been made in understanding the neurobiology underlying impulsivity and aggression, the efficacy of treatments has not been proven.^23^ NICE reviewed the evidence in 2015 and have not amended the recommendations. Therefore, with no clear evidence to support the effectiveness of psychotropic medications for personality disorder, caution is required when prescribing in mental health services and primary care and medication should only be used as an adjunctive treatment, with psychosocial interventions taking the primary role.

Previous research has found there to be a reduced life expectancy in people with borderline personality disorder because of higher risk of suicide.^24,25^ Our findings indicate that quality standards were not consistent across services. Continuity and stability of care is required. Preventing, rather than responding to crises can be achieved by having a consistent approach and building a trusting relationship between staff and patients, ensuring the development of clear crisis planning and facilities, and avoiding in-patient admission and the use of the Mental Health Act where possible. With the rapidly increasing advancement in treatment options for this diagnosis, reducing suicidality in patients with personality disorder is achievable. A clearer pathway for patients to access care is required to guide both clinicians and patients. Dale et al's (2017) national audit of NHS trusts and independent hospitals in England also illustrated this.^5^ Despite an increase in services for personality disorder, there remains variability in patients being able to access them. It is recommended that a further audit of services across the UK be undertaken to evaluate the quality of care provided.

### Strengths and limitations

The key strength of this study is its unique, large and representative sample, collated by The National Confidential Inquiry into Suicide and Safety in Mental Health over a 20-year period. However, the patient numbers studied are from a 1-year consecutive case series across the UK, we acknowledge that this is a snapshot of the most serious incidents, and is not indicative of care for all individuals with personality disorder. We cannot conclude that gaps in care identified reflect the care provided to all patients with borderline personality disorder in the UK. Furthermore, this is a descriptive study and therefore a causal relationship cannot be drawn between patient suicide and the mental health services provided.

It should also be noted that the proportion of patients with a diagnosis of personality disorder who died by suicide in this study, may be lower than reported in other research. This could be the result of the underdiagnoses of personality disorder in clinical practice. To determine clinician's adherence to NICE guidelines prior to suicide, we examined medical records and serious incident reports for patients with personality disorder only. As we have not explored adherence to guidance for other diagnostic groups or patients with personality disorder who did not take their own life, we are unable to make any direct comparisons.

## Data Availability

The authors have not agreed to share this data.
