# The clinical characteristics and care pathways of patients with personality disorder who died by suicide – CORRIGENDUM

**DOI:** 10.1192/bjo.2020.21

**Published:** 2020-04-01

**Authors:** Sandra Flynn, Jane Graney, Thabiso Nyathi, Jessica Raphael, Seri Abraham, Sandeep Singh-Dernevik, Alyson Williams, Nav Kapur, Louis Appleby, Jenny Shaw

**Keywords:** Personality disorder, suicide and self-harm, mental health services

This article states incorrectly that ‘new crisis services were introduced via the Mental Health Act 2007 for people with serious mental illness such as schizophrenia, as short-term interventions’. This line should have stated that ‘new crisis services were introduced via the NHS Plan (2000) for people with serious mental illness such as schizophrenia, as short-term interventions’.
